# A spectroscopic method for monitoring photochemical reactions in the gas phase

**DOI:** 10.1016/j.mex.2022.101847

**Published:** 2022-09-07

**Authors:** Bedabyas Behera, Prasanta Das

**Affiliations:** a Department of Inorganic and Physical Chemistry, Indian Institute of Science, Bangalore - 560012, Karnataka, India; b Department of Chemistry, Mehsana Urban Institute of Sciences, Ganpat University, Ganpat Vidyanagar, Kherva - 384012, Mehsana, Gujarat, India

**Keywords:** Repetitive scan FT-IR spectroscopy, Multi-pass long-path gas cell, Nd:YAG laser(ns), Non-repetitive reaction, Photodegradation of halobenzenes

## Abstract

We have developed an infrared spectroscopic method for monitoring photochemical reactions in the gas phase. This method is based on the major components such as repetitive scan FT-IR spectrometer, multi-pass long-path gas cell, and Nd:YAG laser (ns). The FT-IR spectrometer was used as it is. The gas cell was further modified for the photolysis of the precursor. The vacuum line was designed and constructed solely in our laboratory. We make optical arrangements for both separation of fourth-harmonics (266 nm) from the fundamental (1064 nm) of Nd:YAG laser as well as to guide the UV light to the gas cell housed in the sample compartment of FT-IR. A special arrangement was done in order to get a multi-pass of UV light across the gas cell so that photolysis efficiency will increase significantly. We estimate the photolysis efficiency based on laser power, optical path-length of the laser light, vapor pressure of the precursor, and its absorption cross-section. Furthermore, we have done quantitative analysis for the precursor and photo-products using infrared absorbance and optical path length. This method is tested and validated by monitoring the photodegradation pathways of halobenzenes in the UV light.

• Repetitive scan FT-IR spectrometer coupled with a multi-pass long-path gas cell and Nd:YAG laser.
• Estimate photolysis efficiency of precursor and concentration of photoproducts.
• Monitoring gaseous phase photochemical reactions up-to 100 of ms with spectral resolution 2 cm^−1^ is possible with this system.

## Graphical abstract

**Figure:**
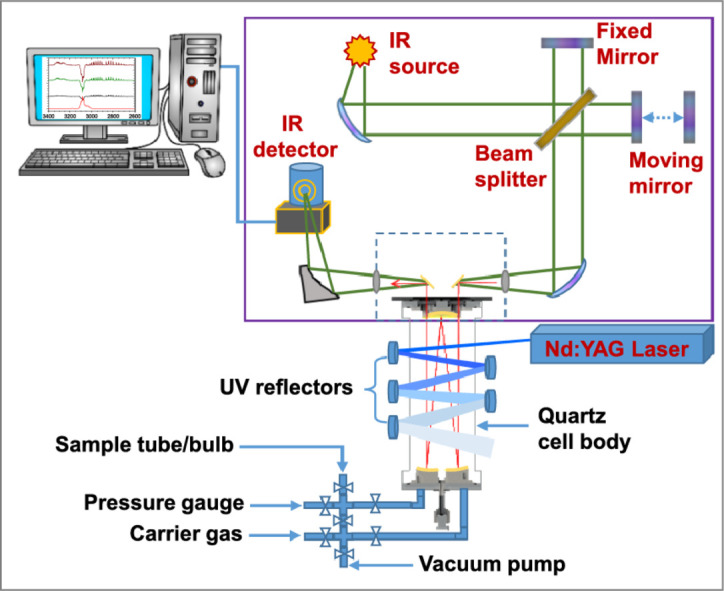

Specifications table

**Table:** 

Subject Area:	Chemistry
More specific subject area:	*Physical chemistry, vibrational Spectroscopy, photochemistry*
Method name:	*Repetitive scan FT-IR spectroscopy for the non-repetitive photochemical reactions*
Name and reference of original method:	*Bedabyas Behera, Prasanta Das, Chem. Phys. Lett.***2021***, 774, 138601*
Resource availability: Tools/reagents	**Equipment** FT-IR spectrometer (Vertex 70, Bruker Optics) Nd:YAG laser (PRO-230, Spectra Physics) Multi-pass long-path gas cell (136G/3TQ, Bruker Optik) Quarter inch fittings and tube (Swagelok) Vacuum pump (Model RV8, BocEdwards) Computer with USB interface **Software** OPUS (Version 7.2, Bruker Optics) GRAMS/AI (Version 7.02, ThermoGalactic) Origin Graphing & Analysis (OriginPro-2018) **Reagent** C_6_H_5_Cl(99.9% HPLC grade), Argon(Ar, UHP), Nitrogen (N_2_, UHP)

## Background

The method we developed for monitoring photochemical reactions is based on Fourier transform infrared (FT-IR) spectroscopy and laser(ns). FT-IR spectrometer is an analytical tool being used to obtain molecular structural information based on spectral signatures of molecular vibrations. It has been employed widely in all branches of chemistry, environmental sciences, molecular biology, chemical and pharmaceutical industries, and many more. Thus became a standard instrument in modern chemical laboratories. Since its discovery, it has been passed through several modifications and made a significant improvement in signal-to-noise ratio, spectral resolution, detection limit, etc. A special effort has been made to reduce the data acquisition time so that molecular changes that happened during reaction can be investigated in-situ [1]. This includes development of time-resolved FT-IR spectroscopy such as rapid scan which covers, typically, the time resolved of 10^2^ - 10^−1^ s [2]. We assemble a repetitive scan FT-IR spectrometer, laser (ns), gas cell, and vacuum lines to study the atmospherically important photochemical reactions in the gas phase. This method possesses several advantages; such as it can measure wide spectral range which allows the detection of many species simultaneously and greater accuracy of wavenumbers. This method can be used to study the chemical evolution within a well-defined isolated system, which is well suited for applied problems. The disadvantages of this technique is that it suffers a lack of sensitivity as compared to techniques available based on molecular beam combining laser and mass spectrometry. However, by the use of a multi-pass long-path gas cell one can improve the sensitivity of this technique. In this report, we present how assembling followed by data acquisition have done. We have also discussed about quantitative estimation of precursor and photoproducts.

## Method details

**Fig. 1:**
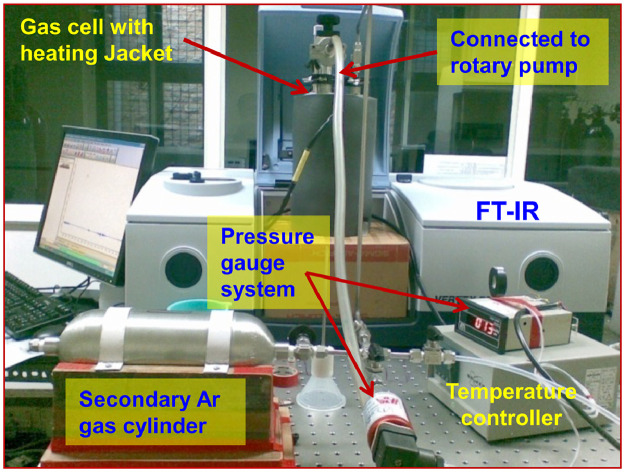
FT-IR spectrometer coupled with multi-pass long-path gas cell with heating jacket and vacuum line.

A part of the experimental set-up shown in Fig. 1 (reproduced from ref. [3]) has been described elsewhere [4], [5], [6]. It consists of FT-IR spectrometer, gas cell, and vacuum line. FT-IR spectrometer (Vertex 70, Bruker Optics) utilized in this set-up was equipped with a liquid-nitrogen-cooled photovoltaic mercury-cadmium-telluride (PV-LN-MCT) detector and a KBr beamsplitter. This set-up was used for investigating vibrational spectra of gaseous polycyclic aromatic hydrocarbons (PAHs), isomeric identification of methylated PAHs, conformational analysis of diols, and isomeric identification of oligomers of alcohol at low concentrations. The higher detection limit is achieved by employing a multi-pass long-path gas cell. This basic set-up has been modified and coupled with UV laser in order to investigate the photochemical reactions in the gas phase [7], to be discussed in the following sections.

**Fig. 2:**
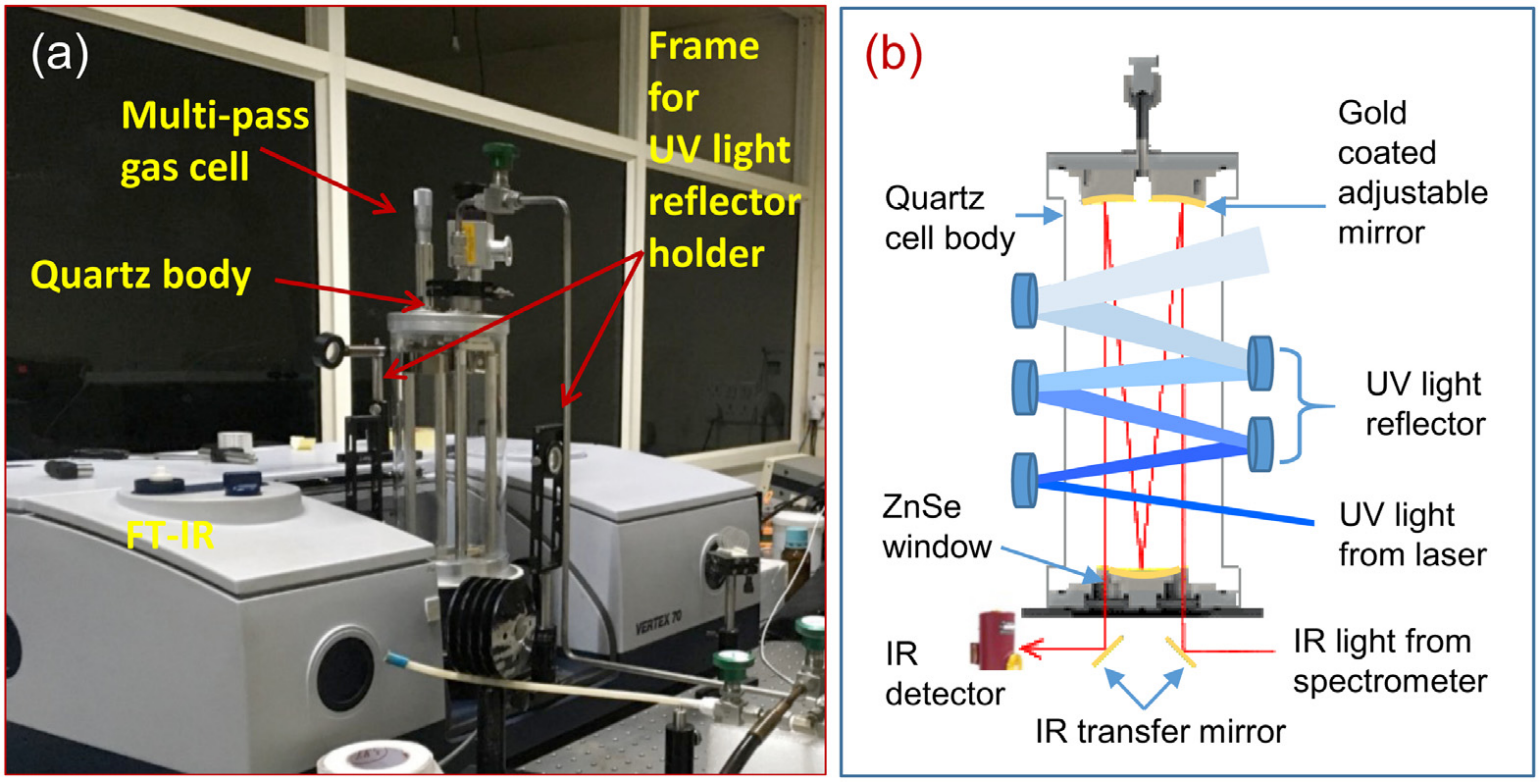
(a) Modified multi-pass long-path gas cell coupled with FT-IR spectrometer. (b) Optical arrangements for UV-light for photolysis and IR light (representative single-pass) for infrared spectroscopic detection.

The gas cell (136G/3TQ, Bruker Optics) shown in Fig. 1 served as a reactor in our experiment was obtained commercially. The gas cell was placed perpendicularly inside the spectrometer sample compartment and connected to the vacuum line. It was made according to the “white cell” principle [8] and contains three gold-coated concave mirrors; two of them are adjustable attached at the top and one is fixed at bottom of the cell. The adjustable mirrors are placed at the radius of curvature of the fixed mirror. The optical path length of the cell can be varied by changing the position of the adjustable mirror. At the entrance and exit point of the IR radiation two ZnSe windows are used. Outside of both the windows two adjustable transfer mirrors are used to guide the IR light from the source to the detector through the cell. Its borosilicate glass body was replaced with a Quartz tube of diameter 11 cm, length 19 cm, and thickness 2 mm to allow UV light for the photolysis, see Fig. 2. The infrared optical path length of the gas cell was set to be 7.2 meters. This is done by adjusting the position of the adjustable mirror and simultaneously counting the He-Ne laser spots on the fixed mirror, see ref [3] for further details. From the number of spots, we can calculate IR optical path length as recommended in the manual (e.g. one spot corresponds to 0.8 meter).

The vacuum line is homemade using 1/4" valves, tubing, and fittings from Swagelok. It is connected to the gas cell, pressure transducer (PRM-300 MX, IRA), vacuum pump (RV8, Boc Edward), Ultra-Torr for the sample tube or bulb, and a gas reservoir cylinder. The sample (solid/liquid) holder is a glass tube/bulb which is attached to the vacuum line using Ultra-Torr fitting and O-ring (Fluorocarbon FKM). The argon (Ar) gas kept in the reservoir cylinder is used for cleaning the cell as well as carrier gas.

**Fig. 3:**
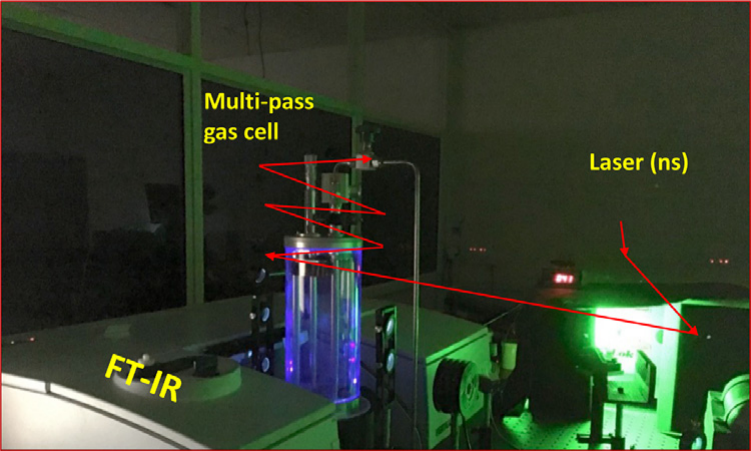
Complete set-up consists of FT-IR, modified gas cell, and Nd:YAG laser.

Fig. 3 shows the complete set-up which was used to investigate UV photodegradation pathways of halobenzenes. The 266 nm light was generated from the fourth harmonic (1064 nm) of a pulse Nd:YAG laser (PRO-230, Spectra Physics). Both the wavelengths of light were separated by a Pellin-Broca prism placed near the laser head. In order to increase the photolysis efficiency, we make a multi-pass arrangement for the laser light as well using five UV reflectors as shown in Figs. (2(b) and 3). The reflectors were placed on circular holders which were hang on a rectangular frame. The frame has a provision for “x” and “y” movements as it can be seen in Figs. (2 and 3). This reflector set-up was placed parallel to the cell body to benefit six times UV light passes through the gas cell. Through this optical arrangement, we cover a UV light irradiation volume of ∼20.3 cm^3^. The laser pulses energy was measured before entering the gas cell to be (37 ± 3) mJ pulse^˗1^. However, inside the cell, the energy is lower due to absorption by cell body material and UV reflectors, which is taken into account in the photolysis yield estimation.

The sample can be loaded in a glass tube or bulb depending on the state of the sample and then attached to the vacuum line through an Ultra-Torr. The dissolved gases are removed by applying several cycles of freeze-pump-thaw. The sample such as chlorobenzene (C_6_H_5_Cl) vapor was first allowed to expand at 298 K into the vacuum line and then transfer to the gas cell and mixed with ∼140 Torr of carrier gas argon (Ar, UHP). We set the amount of precursor after measuring its infrared spectra as a function of sample pressure so as to keep the absorbance below 1.2. Typically, a small amount (<0.5 torr) of sample vapor was loaded in the gas cell for the photolysis studies. We measure partial pressure of sample inside the gas cell from its infrared integrated absorbance, see Section 4. The spectrometer and sample compartment were continuously purged with ultra-high purity (UHP) nitrogen gas during experiments to avoid interference from moisture and carbon dioxide.

We first measured the infrared spectrum of the precursor (e.g. C_6_H_5_Cl) without photolysis with a spectral resolution 2 cm^−1^ and averaged over 128 scans. It takes about 53 second with the conventional measurement of FT-IR. The precursor stability as well as its rate of condensation was tested prior to photolysis by measuring its infrared spectra at different time interval up to several minutes. Then the irradiation was started and simultaneously infrared spectra were recorded. To monitor the reaction, we have acquired several spectra at different photolysis time intervals. Unlike conventional method, the product spectra were recorded using the repeated mode measurements of FT-IR. During this measurement, spectrometer has been set to acquired 5 spectra at 120 sec intervals for a total period of about 9 min.

Point to be noted, the laser light irradiation and repetitive scan measurement were started at the same time manually. The repetitive mode measurement helps us to monitor photolysis products on-line. The precursor spectra were subtracted with proper weighing factors from the spectra recorded during the photolysis to get the product spectra which is called difference spectra. The depletion and formation of infrared bands in the difference spectra indicate the decomposition of reactant and formation of new products, respectively. All the data are processed through OPUS software (version 7.2 Bruker Optics). An additional software GRAMS/AI (version 7.02 Thermo Galactic) was used for the spectral baseline corrections and estimating infrared absorbance for quantitative analysis.

The FT-IR spectrometer can record spectra of wide range of wavenumbers starting from mid (4000 - 400 cm^−1^) to near (12000 - 4000 cm^−1^) infrared. For both the regions separate infrared sources, beamsplitter, detector, and optics combination are used. We have provision for recording spectra in the mid-IR as well as NIR region. The maximum spectral resolution can be recorded up to 0.2 cm^−1^. There is always trade-off to play with spectral resolution and number of scans per spectra to achieve a maximum temporal resolution with a particular spectrometer. In our spectrometer, per scan required 414 ms (since 53 sec is taken for 128 scans) at spectral resolution 2 cm^−1^ over the spectral region 4000 to 700 cm^−1^. Therefore, monitoring a photochemical reactions up-to 100’s of ms with spectral resolution 2 cm^−1^ is possible with this technique.

## Estimation of photolysis efficiency

In this section we discuss the estimation of the photolysis yield which help us to know the efficiency of our experimental set-up. In order to do that we took photolysis of (0.43 ± 0.01) torr C_6_H_5_Cl at 266 nm as an example. The photolysis decay (x) of precursor (C_6_H_5_Cl) is estimated using the following equation

(1) $$x=n\left(C_{6\;}H_{5}Cl\right)\;\times \sigma \left(266nm\right)\times F\;$$

where, n is the amount of C_6_H_5_Cl (PV/RT = 2.829 × 10^17^ molecules) present in the laser active volume (V) 20.3 cm^−3^, F is the laser fluence 13.181 × 10^16^ photon cm^−2^ pulse^−1^ [assuming over all 25% loss on measure energy 37 mJ pulse^−1^ due to cell and UV reflectors absorption and laser active area 0.283 cm^2^ for ∼6 mm laser light beam diameter], and σ is the absorption cross-section of C_6_H_5_Cl (4.933 × 10^−19^ cm^2^ molecule^−1^) which is according to the reported ε(266 nm) = 129 cm^−1^ L mol^−1^ [9]. According to Eq. (1), the amount of photoexcited C_6_H_5_Cl or decay of it's found to be 1.832 × 10^16^ molecules pulse^−1^ in 20.3 cm^3^. Eventually, this much photoproducts get distributed over the cell volume 1.805 × 10^3^ cm^3^ and thus value of x is estimated to be 1.015 × 10^13^ molecules cm^−3^ pulse^−1^ which corresponds to 3.129 × 10^−4^ torr pulse^−1^. The laser is operated at 10 Hz repetition rate and thus overall accumulated decay of precursor during 53 sec photolysis is estimated to be 0.166 torr; that means efficiency is 38.6%. In other words, it's clear that dropping of 0.166 torr C_6_H_5_Cl is the lower limit for observing major photolysis products during first 53 sec. In earlier [7], we established that the photolysis of C_6_H_5_Cl at 266 nm leading to primary process such as C−Cl bond fission and four center concerted HCl elimination. According to energetics, both the channels are equally efficient and thus we can expect 0.083 torr HCl, which is sufficient for the detection using FT-IR spectroscopy to be discussed in the following section.

## Estimation of reactant and photoproduct

**Fig. 4:**
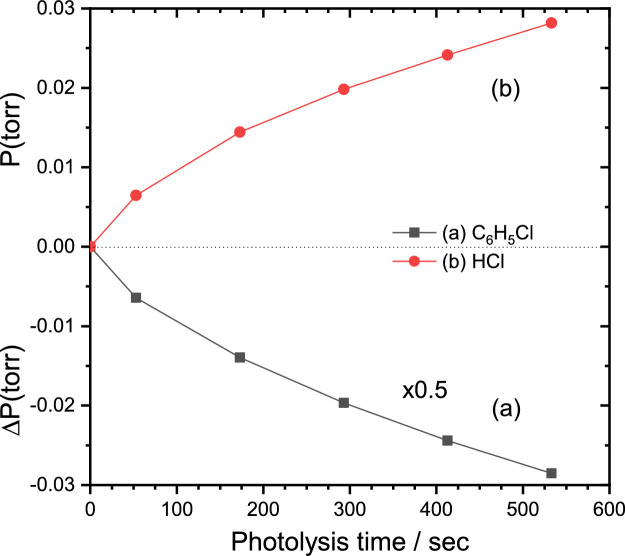
(a) Decay of precursor chlorobenzene (C_6_H_5_Cl) and (b) growth of primary product hydrochloric acid (HCl) [reproduced from Ref. 7].

We measured the decay of sample and formation of products from the integrated band area of the observed infrared spectra using the expression given in ref [10],

(2) $$P_{i}\left(atm\right)=\frac{2.303\;\times \;82.05\;\times \;T\times 10^{-5}\times \;\tau_{i}}{A_{i}\times l},\;$$

in which, *τ_i_* is the integrated absorbance (in cm^−1^), $A_{i}$ (in km mol^−1^) is the absorption coefficient, *l* (in cm) is the optical path length, and T (298 K) is the experimental temperature. Typically, we chose 2 - 3 absorption lines of a species and averaged the derived partial pressure. In case of rotational-resolved spectra, sum of band areas over all resolved lines is assumed to be equivalent to the unresolved band area within error limit. The derived pressure errors are typically within ± 20% due to the fitting errors. According to the measured infrared absorbance, the initial pressure of precursor is estimated to be 0.427 ± 0.013 torr. During 53 s photolysis the concentration of C_6_H_5_Cl is decreased by (0.0128 ± 0.241 × 10^−3^) torr and formation of primary product HCl is estimated to be (0.00645 ± 0.19894 × 10^−4^) torr as reflected in Fig. 4. This is ∼13 times less as compared to laser photolysis yield. This clearly indicates, perhaps, some of the products are absorbed by the cell component.

## Test output

**Fig. 5:**
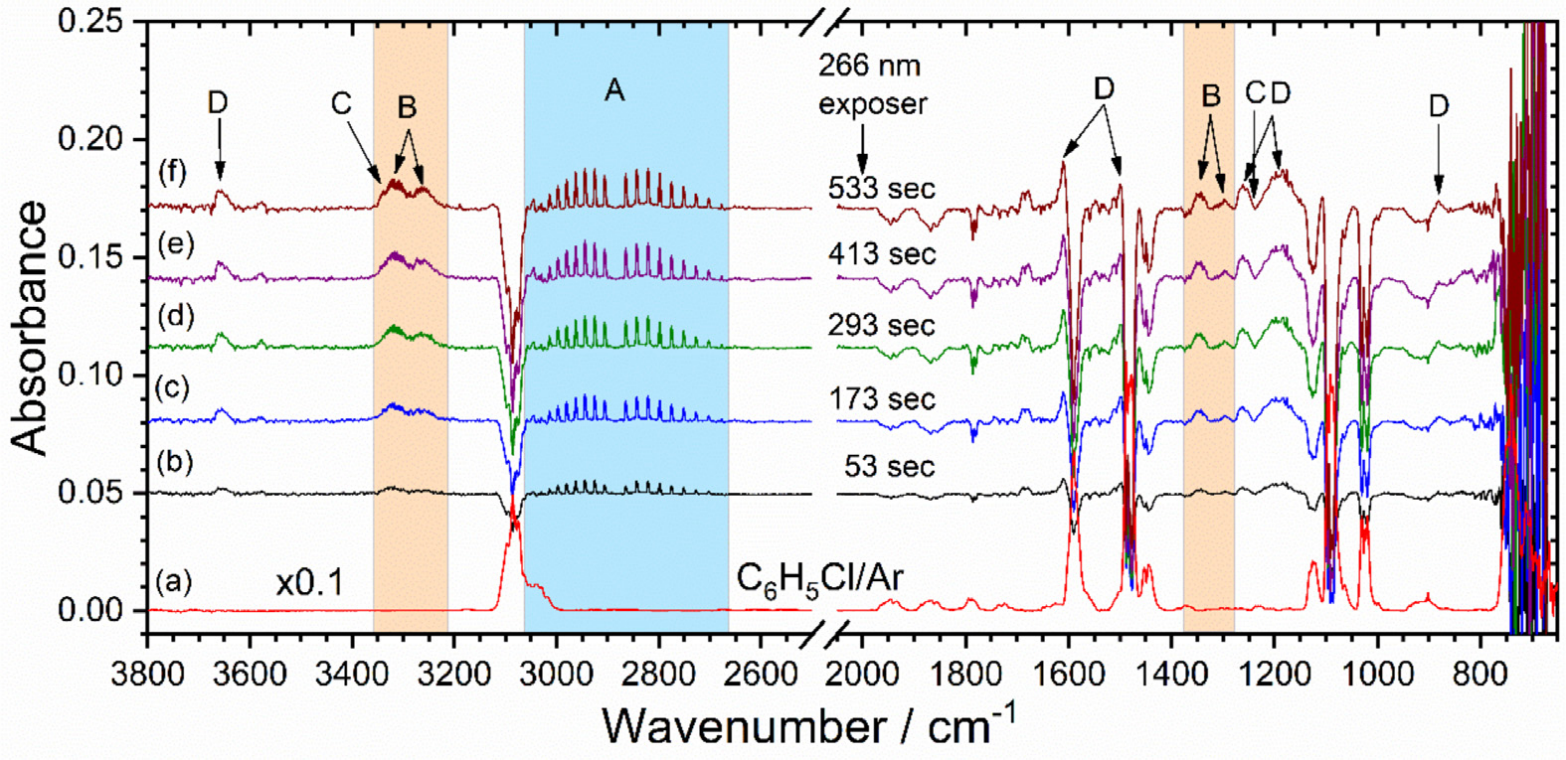
(a) Infrared absorption spectra of 0.43 ± 0.01 torr C_6_H_5_Cl seeded in 142 torr argon (Ar). Difference spectra (b - f) were measured during 53, 173, 293, 413, and 533 sec photolysis at 266 nm. Infrared absorption band indicated by A, B, C, and D are assigned to hydrochloric acid (HCl), acetylene (C_2_H_2_), 1,3-butadiyne (C_4_H_2_), and phenol (C_6_H_5_OH), respectively, see main text of ref. [7] for the detail assignment.

**Fig. 6:**
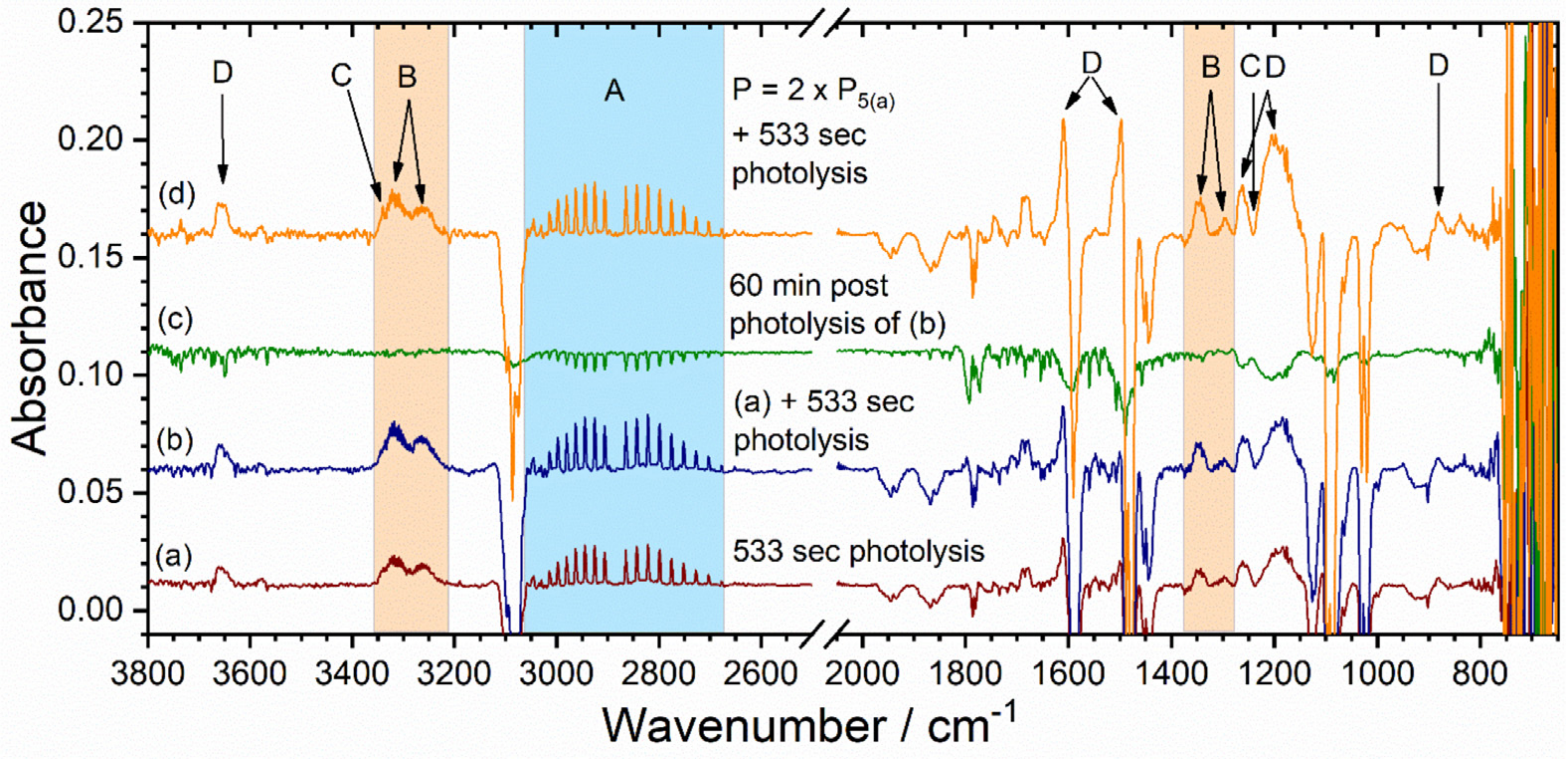
(a) Reproduced Fig. 5(f) for comparison. (b) Difference spectra obtained after additional 533 sec photolysis of (a). (c) Difference spectra obtained after 1 h post photolysis of (b) minus spectra (b). (d) Difference spectra obtained in another day with double amount of precursor (C_6_H_5_Cl). P_5(a)_ is the pressure of C_6_H_5_Cl loaded for spectra 5(a). For the assignment of species A, B, C, and D see the caption of Fig. 5 and main test of ref [7].

The above described method was tested and validated upon successful photodegradation studies of C_6_H_5_Cl [7]. Fig. 5(a) shows the spectra of precursor (C_6_H_5_Cl) which is obtained based on normal scan measurement, whereas, spectra in Fig. 5(b - f), obtained during 53, 173, 293, 413, and 533 sec photolysis of C_6_H_5_Cl were based on repeated measurement. Details assignment of photodegraded products and reaction mechanism has been discussed in our recent publications [7]. In some instances, we have done the test to see the effect of prolonged photolysis; Fig. 6 (b) showing the spectra of C_6_H_5_Cl under such conditions, which indicates that upon long time irradiation both the primary and secondary products go up. Fig. 6(c) shows 1-hour post photolysis spectra of C_6_H_5_Cl. We can see that IR features for both products and reactant slightly goes down and there are no new features coming up. This clearly indicates that there is no or undetectable new products formation upon keeping the post photolysis mixture. When photolysis was carried out with the double amount of precursor, we observed no additional IR features but those for secondary photoproducts (e.g. group D lines which are assigned to phenol) go up significantly, see Fig. 6(a) and (d). In short, reproducibility in terms of observing species are satisfying well.

We have successfully applied this method for investigating photodegradation of C_6_H_5_Br at 266 nm. This repeated mode measurement can be applied to monitor the thermal and photo-degradation process not only in gas phase but also solid and liquid samples using a suitable cell set-up.

## Declaration of interests

The authors declare that they have no known competing financial interests or personal relationships that could have appeared to influence the work reported in this paper.
